# J-Shaped relationship between the red cell distribution width to albumin ratio and erectile dysfunction: a cross-sectional study from NHANES 2001-2004

**DOI:** 10.3389/fendo.2025.1545272

**Published:** 2025-03-25

**Authors:** Yang Xu, Shuofeng Li

**Affiliations:** ^1^ Department of Urology, Huzhou Central Hospital, Fifth School of Clinical Medicine of Zhejiang Chinese Medical University, Huzhou, China; ^2^ Department of Urology, Huzhou Central Hospital, Affiliated Central Hospital of Huzhou University, Huzhou, China; ^3^ Department of Urology, Affiliated Hospital of Xuzhou Medical University, Xuzhou, China

**Keywords:** erectile dysfunction, red cell distribution width, J-shaped association, NHANES, cross-sectional study

## Abstract

**Background:**

Erectile dysfunction (ED) is a prevalent condition closely associated with systemic inflammation and metabolic disorders. The red cell distribution width to albumin ratio (RAR) is an emerging inflammatory marker; however, its relationship with ED remains poorly understood.

**Methods:**

This study conducted a cross-sectional analysis of data from 3,950 participants in the National Health and Nutrition Examination Survey (NHANES) 2001–2004 cycle to evaluate the association between RAR and ED risk. A Multivariable logistic regression model was employed to assess the relationship between RAR and ED, while a generalized additive model (GAM) and dose-response analysis were utilized to explore potential nonlinear associations. Subgroup analyses were performed to investigate interactions with demographic and lifestyle factors.

**Results:**

Among the study population, 1,157 individuals reported a history of ED. The prevalence of ED was significantly higher in individuals aged 50 years and older (86.78%) and was associated with increased rates of hypertension, diabetes mellitus, and cardiovascular disease (*P* < 0.001). A J-shaped relationship was identified between RAR and ED risk. Specifically, the risk of ED significantly increased below the RAR threshold of 3.42 (OR = 3.01, 95% CI: 2.08–4.36, *P* < 0.001), while the risk plateaued at higher RAR values. Subgroup analyses revealed significant interactions with ethnicity (P = 0.018) and moderate-intensity physical activity (*P* = 0.004). Non-Hispanic whites (OR = 2.85) and individuals engaging in moderate-intensity activity (OR = 3.83) exhibited a heightened risk of ED. No significant interactions were observed for other variables, including age and BMI.

**Conclusion:**

The results demonstrated that RAR was independently associated with ED risk, exhibiting a J-shaped relationship. There was a significant increase in risk below RAR = 3.42, with saturation occurring after exceeding this threshold.

## Introduction

Erectile dysfunction (ED) is a prevalent chronic condition defined by the inability to obtain or maintain an erection sufficient for the completion of satisfactory sexual activity (1). As the population continues to age, the incidence of ED is rising, with a notable impact on the quality of life and mental health of men (2). Concurrently, ED is not merely an expression of a local pathological process; it is also regarded as a comprehensive manifestation of systemic diseases, with a strong correlation to systemic inflammation and metabolic disorders (3). Prior research has demonstrated a significant association between ED and chronic illnesses such as cardiovascular disease and diabetes, underscoring its potential as a marker of systemic diseases (4, 5).

In recent years, there has been a notable increase in the number of studies examining the role of inflammatory markers in the context of chronic diseases (6, 7). Red cell distribution width (RDW), a sensitive indicator of blood inflammation and oxidative stress, has been demonstrated to be significantly associated with the risk of cardiovascular disease, diabetes, and metabolic syndrome (8–10). Concurrently, serum albumin level, as a crucial indicator of nutritional status and anti-inflammatory function, also plays a pivotal role in the investigation of inflammation-related diseases (11, 12). However, the predictive capacity of these individual indicators may be constrained (13). The RDW to Albumin Ratio (RAR) has recently emerged as a promising composite marker, integrating the advantages of RDW and albumin to provide a more comprehensive reflection of systemic inflammatory levels and nutritional status (14, 15). The predictive value of RAR in the risk assessment of cardiovascular disease and diabetes has been demonstrated in numerous studies (16, 17). However, its potential association with ED remains to be elucidated.

To address this gap, we conducted a nationally representative cross-sectional study using NHANES 2001–2004 data. Our analysis focused on adult males, leveraging standardized laboratory measurements and validated questionnaires to examine the RAR-ED relationship while adjusting for sociodemographic, metabolic, and lifestyle confounders.

## Methods

### Study population

The data for this study were derived from the NHANES, conducted by the National Center for Health Statistics (NCHS). NHANES is a nationwide population-based cross-sectional study of non-institutionalized U.S. citizens, the objective of which is to assess their nutritional status and potential health risk factors. In order to ensure the representativeness of the sample, a complex hierarchical and multi-stage probabilistic cluster sampling design was adopted. The study protocol for NHANES has been approved by the NCHS Research Ethics Review Committee, and all participants have provided written informed consent. The specific study design and data for NHANES are accessible via the CDC website (www.cdc.gov/nchs/nhanes/).

**Figure 1:**
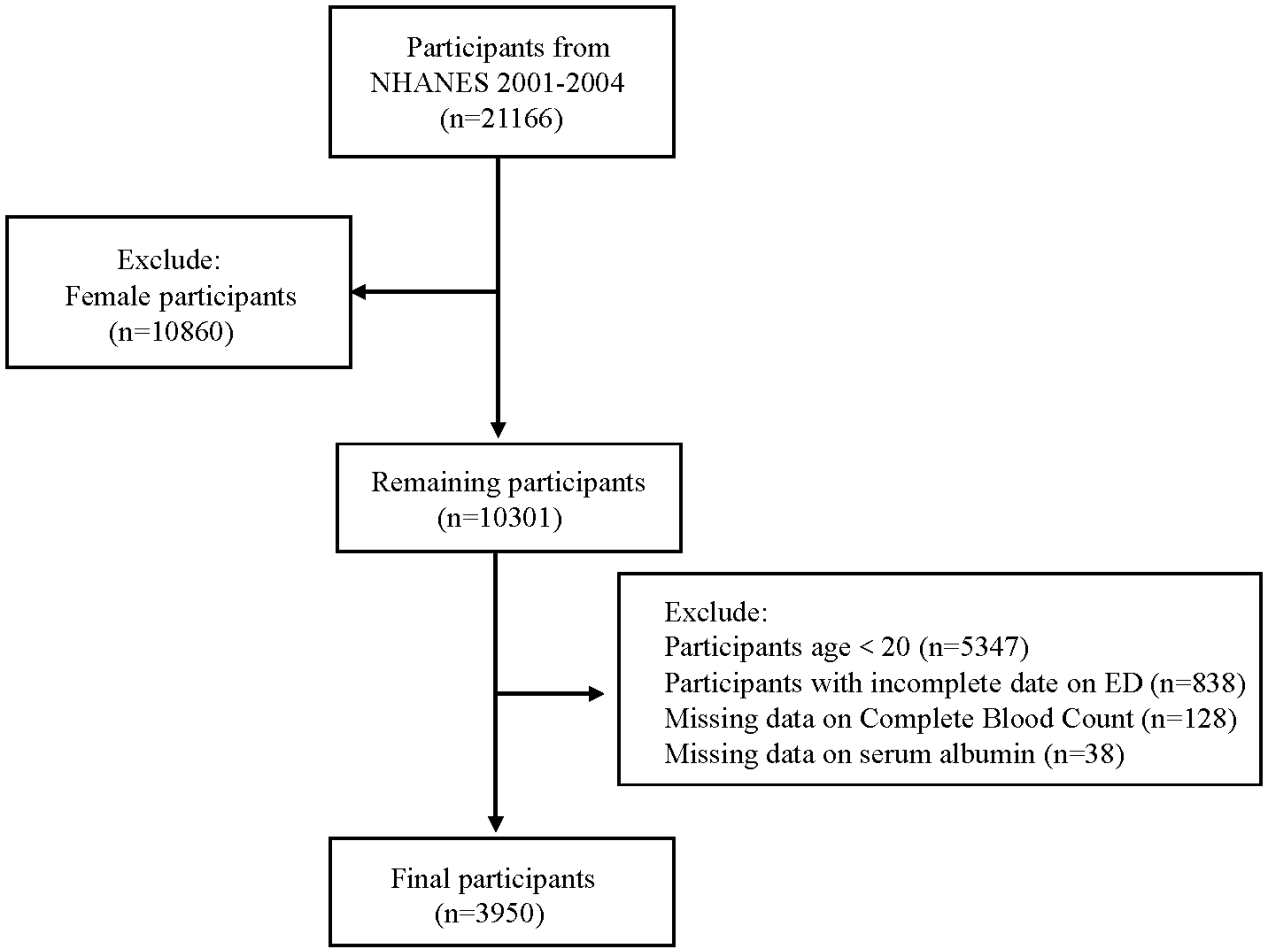
Flowchart of participant selection process of NHANES 2001-2004.

We analyzed data from the NHANES 2001–2004 cycles (2001–2002 and 2003–2004), which were the only surveys including ED-related questionnaires. Initially, 21,161 participants were screened. After applying exclusion criteria—(1) female sex (n = 10,860), (2) age <20 years (n = 5,347), (3) missing ED data (n = 838), (4) missing RDW values (n = 128), and (5) missing serum albumin levels (n = 38)—a final cohort of 3,950 participants was included (**Figure 1**).

### Assessment of RAR and ED

Blood samples are collected at Mobile Health Examination Centres (MECs) as part of the NHANES. The RDW is determined by a Beckman Coulter MAXXM instrument as part of the complete blood count. The instrument employs the Beckman Coulter method for counting, measuring size, and automating dilution and mixing to process samples (18). Serum albumin concentrations were determined using the DcX800 method, which employs a two-color ratio digital endpoint method to form a complex with serum albumin utilising bromocresol violet (BCP) reagent (19). The RAR is calculated as the ratio of RDW to albumin. In the NHANES database, the question that assesses erectile function is as follows: “During this period, how would you rate your ability to achieve and maintain an erection that is sufficient to make a satisfactory erection?” The response options are as follows: (1) “Never able to get and keep an erection,” (2) “Sometimes able to get and keep an erection,” (3) “Usually able to get and keep an erection,” and (4) “Always or almost always able to get and keep an erection.” Participants selecting options (1) or (2) were classified as having ED, while those choosing options (3) or (4) were categorized as non-ED. Those who selected the first two options were diagnosed with ED, while those who selected the latter two were defined as having no ED. Furthermore, the validity of self-reported methods for diagnosing ED has been validated (20).

### Covariates

In the present study, the covariates included the following: (1) Basic demographic variables, including age, ethnicity, education level, marital status, poverty-to-income ratio (PIR), and (Body Mass index) BMI, were also considered. (2) Lifestyle variables, including smoking, alcohol intake, and physical activity, The ethnic backgrounds were divided into the following categories: Mexican-American, Non-Hispanic White, Non-Hispanic Black, Other Hispanic, and Other Races. The educational levels are classified into three categories: below high school, high school, and above. The marital status is classified as never married, widowed/divorced/separated, and married/living with a partner. The PIR, which serves as an indicator of economic status, is divided into three categories. PIR ≤ 1.3, 1.3–3.5, and >3.5. The term “non-smoker” is used to describe men who have never smoked more than 100 cigarettes in their lifetime. The term “alcohol intake” is defined as the consumption of at least 12 alcoholic beverages over the past 12 months. The diagnosis of medical complications, including diabetes, cardiovascular disease (CVD), and hypertension, is based on self-reported medical history as outlined in the NHANES questionnaire (21).

### Statistical analysis

All statistical analyses were conducted in accordance with the guidelines set forth by the Centers for Disease Control and Prevention (CDC), with the appropriate NHANES weights employed to account for the intricate multi-stage stratified sampling designs. Missing values in covariates were addressed using imputation. In the baseline feature table, categorical variables are expressed as percentages, and continuous variables are summarised as the mean and standard error (SE). The differences between the ED and non-ED groups were assessed using a weighted chi-square test (for categorical variables) or a weighted Student’s t-test (for continuous variables). To investigate the relationship between RAR and the incidence of ED, three logistic regression models were employed. Model 1 is an unadjusted model. Model 2 was adjusted for age, ethnicity, education level, marital status, and PIR. Model 3 was further adjusted for additional variables, including BMI, alcohol intake, hypertension, diabetes, CVD, vigorous activity, moderate activity, smoking status, low-density lipoprotein cholesterol (LDL-C), high-density lipoprotein cholesterol (HDL-C), fasting glucose, triglycerides, and total cholesterol (TC), in accordance with the adjustments made in Model 2. The relationship between RAR and ED was described by means of a weighted Multivariable logistic regression, which was analysed separately as a continuous variable and a categorical variable (tertiary classification).

Subsequently, we conducted a further analysis of the nonlinear relationship between RAR and the prevalence of ED using a generalized additive model (GAM) and smooth curve fitting. In instances where a nonlinear relationship was identified, a two-segment linear regression model (piecewise regression model) was applied to each interval. This was then compared with the unilinear model (non-segmented model) to ascertain the threshold effect, which was calculated using the log-likelihood ratio test. Subsequently, subgroup analyses were performed using stratified logistic regression models. The modifications and interactions of subgroups were inspected by likelihood ration tests.

All analyses were conducted using R software, version 3.4.3 (http://www.r-project.org, The R Foundation), and EmpowerStats software (http://www.empowerstats.com; X&Y solutions, Inc., Boston, MA). The level of statistical significance was set at *P* < 0.05. The p-value of the interaction is employed to indicate the degree of significance of the interaction between the covariate and the primary exposure variable on the outcome variable.

## Results

### Characteristics of participants

**Table 1** presents the baseline characteristics of the 3,950 participants, of whom 1,157 reported experiencing ED. The majority of patients with ED were aged 50 years or above (86.78%), while only a third of patients without ED fell into this age category (*P* < 0.001). The prevalence of hypertension (52.29% vs. 22.84%), diabetes (22.73% vs. 5.33%) and CVD (28.61% vs. 6.12%) was significantly higher in patients with erectile dysfunction (*P* < 0.001). Furthermore, they were less likely to engage in vigorous activity (16.34% vs. 40.49%) and exhibited higher fasting glucose levels (119.69 ± 1.58 vs. 104.45 ± 0.69 mg/dL, *P* < 0.001). Furthermore, patients with ED exhibited lower levels of education and socioeconomic status, a higher proportion of individuals with less than a high school education (40.19% vs. 23.17%), and a higher proportion of individuals with PIR ≤1.3 (28.26% vs. 22.70%, *P* < 0.001).

**Table 1:** Baseline characteristics of the study population.

Characteristic	Total	Erectile dysfunction		P-value
		No	Yes	
Total patients	3950	2793	1157	
Age, year				<0.001
<50	2038 (51.59%)	1885 (67.49%)	153 (13.22%)	
≥50	1912 (48.41%)	908 (32.51%)	1004 (86.78%)	
Race				<0.001
Mexican American	811 (20.53%)	576 (20.62%)	235 (20.31%)	
Other Hispanic	139 (3.52%)	98 (3.51%)	41 (3.54%)	
Non-Hispanic White	2158 (54.63%)	1475 (52.81%)	683 (59.03%)	
Non-Hispanic Black	722 (18.28%)	549 (19.66%)	173 (14.95%)	
Other Race	120 (3.04%)	95 (3.40%)	25 (2.16%)	
Marital status				<0.001
Married/Living with a partner	2715 (68.73%)	1864 (66.74%)	851 (73.55%)	
Widowed/Divorced/Separated	554 (14.03%)	325 (11.64%)	229 (19.79%)	
Never Married	681 (17.24%)	604 (21.63%)	77 (6.66%)	
Educational level				<0.001
Below high school	1112 (28.15%)	647 (23.17%)	465 (40.19%)	
High school	974 (24.66%)	736 (26.35%)	238 (20.57%)	
Above high school	1864 (47.19%)	1410 (50.48%)	454 (39.24%)	
PIR				<0.001
≤ 1.3	961 (24.33%)	634 (22.70%)	327 (28.26%)	
1.3-3.5	1547 (39.16%)	1047 (37.49%)	500 (43.22%)	
≥ 3.5	1442 (36.51%)	1112 (39.81%)	330 (28.52%)	
BMI, kg/m^2^				0.355
< 25	1203 (30.46%)	851 (30.47%)	352 (30.42%)	
25–30	1732 (43.85%)	1241 (44.43%)	491 (42.44%)	
≥ 30	1015 (25.70%)	701 (25.10%)	314 (27.14%)	
Alcohol intake				0.004
Yes	3270 (82.78%)	2343 (83.89%)	927 (80.12%)	
No	680 (17.22%)	450 (16.11%)	230 (19.88%)	
Hypertension				<0.001
Yes	1243 (31.47%)	638 (22.84%)	605 (52.29%)	
No	2707 (68.53%)	2155 (77.16%)	552 (47.71%)	
Diabetes				<0.001
Yes	412 (10.43%)	149 (5.33%)	263 (22.73%)	
No	3481 (88.13%)	2611 (93.48%)	870 (75.19%)	
Borderline	57 (1.44%)	33 (1.18%)	24 (2.07%)	
CVD				<0.001
No	3448 (87.29%)	2622 (93.88%)	826 (71.39%)	
Yes	502 (12.71%)	171 (6.12%)	331 (28.61%)	
Vigorous activity				<0.001
No	2630 (66.58%)	1662 (59.51%)	968 (83.66%)	
Yes	1320 (33.42%)	1131 (40.49%)	189 (16.34%)	
Moderate activity				<0.001
No	2012 (50.94%)	1342 (48.05%)	670 (57.91%)	
Yes	1938 (49.06%)	1451 (51.95%)	487 (42.09%)	
Smoking status				<0.001
< 100 cigarettes lifetime	2411 (61.04%)	1589 (56.89%)	822 (71.05%)	
≥ 100 cigarettes lifetime	1539 (38.96%)	1204 (43.11%)	335 (28.95%)	
LDL-C, mg/dL	119.80 ± 0.91	120.78 ± 1.04	117.43 ± 1.28	0.030
TC, mg/dL	199.68 ± 1.09	200.98 ± 1.15	196.54 ± 2.10	0.004
Triglycerides, mg/dl	165.34 ± 3.95	166.95 ± 4.41	161.45 ± 7.65	0.342
Fasting glucose, mg/dl	108.91 ± 0.60	104.45 ± 0.69	119.69 ± 1.58	<0.001
HDL-C, mg/dL	47.62 ± 0.30	47.63 ± 0.33	47.60 ± 0.47	0.947
RAR	2.96 ± 0.01	2.88 ± 0.01	3.14 ± 0.01	<0.001

PIR, Poverty income ratio; BMI, body mass index; CVD, cardiovascular disease; LDL-C, low-density lipoprotein cholesterol; TC, total cholesterol; HDL, high-density lipoprotein; RAR, red blood cell distribution width to Albumin Ratio.

### Association between RAR and ED

**Figure 2:**
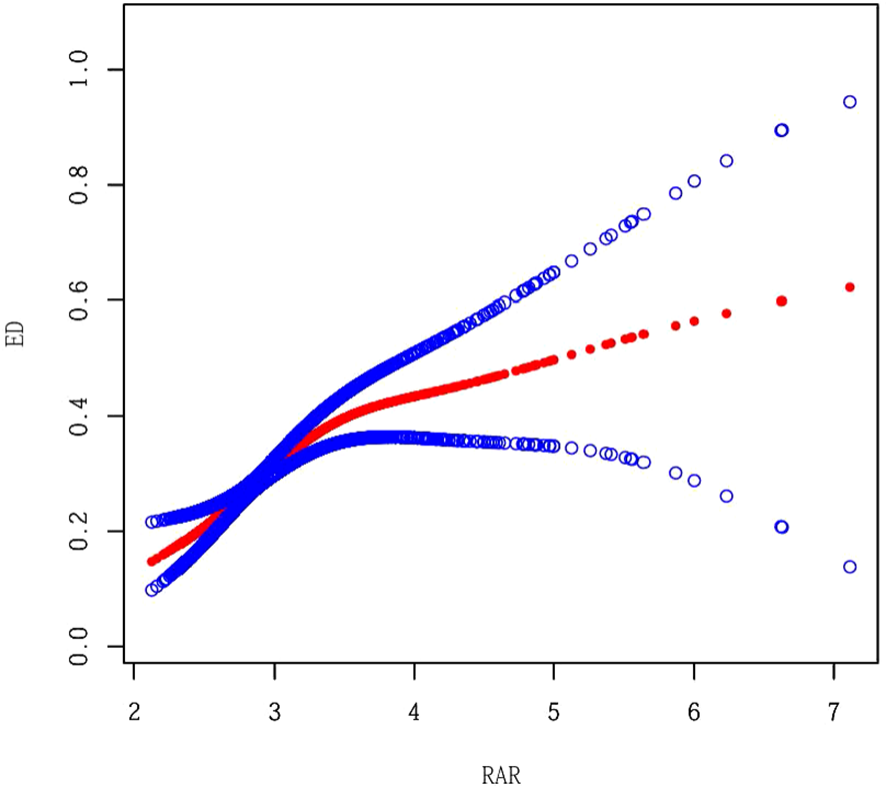
Smooth Curve Fitting between RAR and ED. Adjusted for Age, Race, Marital Status, Education level, PIR, BMI, Alcohol intake, Hypertension, Diabetes, CVD, Vigorous activity, Moderate activity, Smoking status, LDL_C, TC, Triglycerides, DAsting glucose and HDL-C.

As illustrated in **Table 2**, there is a notable correlation between RAR and ED. In all models, a higher RAR was significantly associated with an increased risk of ED. A stratified analysis by RAR quintile demonstrated a dose-response relationship between the high group and a significantly increased risk of ED (*P* for trend < 0.001). Furthermore, we investigated the non-linear relationship between RAR and the prevalence of ED utilising GAM and smooth curve fitting (**Figure 2**). The analysis demonstrated a nonlinear positive correlation between RAR and ED prevalence, with a significantly enhanced association between RAR and ED below the threshold (RAR < 3.42) (OR = 3.01, 95% CI: 2.08–4.36, *P* < 0.001), and a log-likelihood ratio test *P*-value < 0.05 (**Table 3**).

**Table 2:** Association between RAR and ED.

	Model 1	Model 2	Model 3
	OR (95%CI)	OR (95%CI)	OR (95%CI)
RAR	5.81 (4.71, 7.17)	2.52 (2.01, 3.17)	1.92 (1.51, 2.42)
Stratified by RAR tertiles			
Tertile 1 (≤2.77)	1.0	1.0	1.0
Tertile 2 (2.77-3.02)	2.45 (2.01, 2.99)	1.57 (1.25, 1.97)	1.52 (1.20, 1.92)
Tertile 3 (≥3.02)	5.40 (4.46, 6.54)	2.49 (1.98, 3.12)	2.09 (1.65, 2.65)
*P* for trend	<0.001	<0.001	<0.001

Model 1: No covariates were adjusted. Model 2: Adjusted for Age, Race, Marital status, Education level and PIR. Model 3: Adjusted for Age, Race, Marital status, Education level, PIR, BMI, Alcohol intake, Hypertension, Diabetes, CVD, Vigorous activity, Moderate activity, Smoking status, LDL-C, TC, Triglycerides, Fasting glucose and HDL-C.

**Table 3:** Analysis of the threshold effect between RAR and ED.

ED	RAR
	OR (95% CI)
Fitting by standard linear model	1.92 (1.51, 2.42)
*P*-value	<0.001
Fitting by two-piecewise linear model	
Breakpoint(K)	3.42
OR1 < K	3.01 (2.08, 4.36) <0.001
OR2 > K	1.10 (0.74, 1.63) 0.630
Logarithmic likelihood ratio test P-value	0.002

Adjusted for Age, Race, Marital status, Education level, PIR, BMI, Alcohol intake, Hypertension, Diabetes, CVD, Vigorous activity, Moderate activity, Smoking status, LDL-C, TC, Triglycerides, Fasting glucose and HDL-C.

### Subgroup analysis

**Figure 3:**
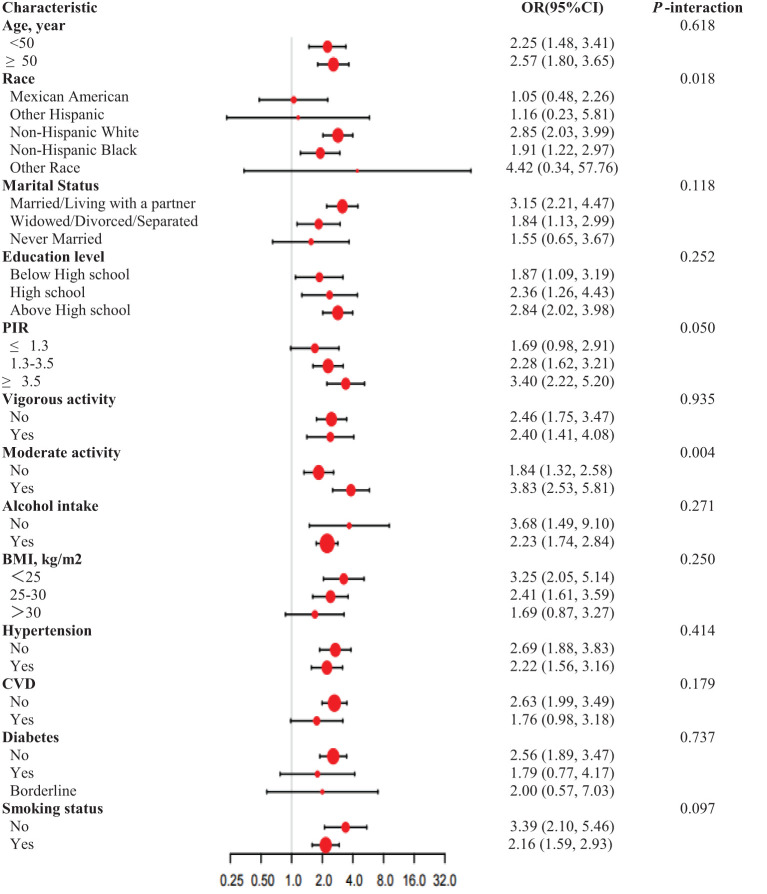
Subgroup analysis for RAR and ED, weighted. Adjusted for Age, Marital status, Education level. PIR, BMI, Alcohol intake, Hypertension, Diabetes, CVD, Vigorous activity, Moderate activity, Smoking status, LDL-C, TC, Triglycerides, fasting glucose and HDL-C. In each case, the model is not adjusted for the stratification variable.

In order to evaluate the consistency of the relationship between RAR and the prevalence of ED in different populations, we conducted subgroup analyses (**Figure 3**). These analyses demonstrated that race exhibited significant interaction effects (*P* = 0.018), with a higher risk observed among non-Hispanic whites (OR = 2.85) and non-Hispanic blacks (OR = 1.91). The interaction effect of moderate-intensity activity was statistically significant (*P* = 0.004), indicating that the risk of moderate activity was increased (OR = 3.83). No significant interactions were observed for other factors, including age, marital status, education level, PIR, vigorous activity, alcohol intake, BMI, hypertension, CVD, diabetes, and smoking status.

## Discussion

This study revealed, for the first time, a nonlinear J-shaped relationship between RAR and ED, offering novel insights into the interplay of systemic inflammation, metabolic dysregulation, and vascular dysfunction in ED pathogenesis. Below the threshold of RAR = 3.42, ED risk increased sharply (OR = 3.01), plateauing beyond this point. This biphasic association suggests distinct pathophysiological mechanisms dominate at different RAR ranges, mediated by the dual roles of RDW and albumin in inflammatory and vascular homeostasis.

At lower RAR levels (<3.42), the heightened ED risk likely stems from synergistic effects of hypoalbuminemia and elevated RDW. Hypoalbuminemia reflects malnutrition or chronic inflammation, impairing endothelial nitric oxide (NO) synthesis—a cornerstone of erectile function—and reducing testosterone bioavailability due to disrupted steroid hormone transport (22, 23). Albumin’s antioxidant properties mitigate oxidative stress; its deficiency exacerbates endothelial dysfunction by permitting unchecked reactive oxygen species (ROS) accumulation (24). Concurrently, elevated RDW signifies erythrocyte heterogeneity driven by inflammation or oxidative stress, which correlates with endothelial damage and impaired microvascular perfusion (25). Studies demonstrate that RDW elevation is linked to reduced NO-mediated vasodilation, a critical defect in ED pathophysiology (26). These pathways collectively create a “perfect storm” for ED at low RAR values, where inflammation and nutritional deficits converge to disrupt vascular and hormonal homeostasis.

Above the threshold (RAR >3.42), the risk plateau may reflect compensatory adaptations to chronic inflammation. Prolonged inflammatory states activate counter-regulatory mechanisms, such as upregulation of anti-inflammatory cytokines (e.g., IL-10) and endogenous antioxidant systems, which partially offset endothelial damage (27). Additionally, chronic hypoxia may stimulate angiogenesis via VEGF, improving collateral blood flow to penile tissues despite systemic inflammation (28, 29). However, this compensatory capacity likely has limits, explaining the attenuated risk escalation at higher RAR levels. Notably, unmeasured confounders, such as subclinical liver disease or micronutrient deficiencies (e.g., iron, vitamin B12), could influence both RDW and albumin, potentially confounding the observed nonlinearity (30). Future studies should address these factors to refine mechanistic understanding.

RAR serves as a composite biomarker that integrates both inflammatory (RDW) and nutritional (albumin) components, providing a more comprehensive assessment of systemic health compared to using these markers individually. Unlike RDW alone, which primarily reflects erythrocyte variability, or albumin, which indicates nutritional status, RAR captures the interplay between inflammation, oxidative stress, and vascular health (31, 32). This dual-component nature enhances its sensitivity in identifying ED risk, particularly in populations with metabolic disorders (33).

Furthermore, the J-shaped association identified in this study suggests that RAR provides superior predictive value over conventional inflammatory markers such as C-reactive protein (CRP) and neutrophil-to-lymphocyte ratio (NLR), which typically show linear associations with disease risk (34). The nonlinear relationship implies that RAR not only identifies high-risk individuals at extreme values but also highlights a potential threshold for early intervention (35). This is particularly relevant for ED, where early detection of vascular dysfunction can facilitate timely lifestyle or pharmacological interventions.

The complexity of RAR as a composite biomarker is further underscored by subgroup analyses. The heightened ED risk among non-Hispanic Whites and physically active individuals suggests that genetic polymorphisms in inflammatory mediators (e.g., CRP, IL-6) may predispose certain ethnic groups to RAR-driven ED (36). Paradoxically, moderate exercise—though generally protective—may transiently exacerbate oxidative stress in susceptible individuals, amplifying ED risk in high-RAR subgroups (37). These interactions highlight the need for personalized approaches, integrating genetic, lifestyle, and inflammatory profiling into ED management.

The J-shaped RAR-ED association has substantial clinical relevance. Since RAR is calculated from routine blood tests (RDW and albumin), it provides a cost-efficient biomarker for ED risk stratification, especially in aging populations or those with metabolic syndrome. Compared to other inflammatory markers, RAR’s accessibility and reliability make it a practical addition to standard ED screening tools. Integrating RAR with the Sexual Health Inventory for Men (SHIM) questionnaire could enhance early detection and improve patient outcomes. For patients with RAR <3.42, targeted strategies—such as anti-inflammatory diets, albumin supplementation, or lifestyle modifications—may mitigate ED progression.

Beyond its localized symptoms, ED is increasingly recognized as a harbinger of systemic pathologies, including cancer and accelerated aging. Recent studies associate ED with cancer progression, possibly via shared pathways of chronic inflammation and oxidative stress, which drive both endothelial dysfunction and genomic instability (38). Furthermore, ED correlates with aging biomarkers such as telomere shortening and senescent cell accumulation, suggesting a role in biological aging (39). These findings position ED not merely as a urological condition but as a multisystem indicator, necessitating holistic management to address underlying comorbidities.

While this study demonstrates a nonlinear relationship between RAR and ED, several limitations must be acknowledged. Firstly, the cross-sectional design precludes causal inference; longitudinal cohorts are needed to validate RAR’s predictive value and establish intervention thresholds. Secondly, missing data in some cases may have introduced potential biases despite statistical adjustments. Additionally, the sample predominantly comprised US individuals, limiting generalizability to other racial and regional groups. Future studies should investigate the molecular mechanisms linking RAR to inflammatory markers, vascular endothelial function, and testosterone metabolism, while evaluating RAR’s clinical utility in diverse populations.

## Conclusion

This study identifies a J-shaped relationship between RAR and ED, implicating inflammation, vascular dysfunction, and compensatory mechanisms in its etiology. RAR’s simplicity and affordability position it as a promising biomarker for ED risk assessment, particularly when combined with aging and systemic disease markers. Future research should prioritize mechanistic studies to unravel RAR’s role in endothelial and hormonal pathways, alongside randomized trials testing RAR-guided interventions.

## Data Availability

The raw data supporting the conclusions of this article will be made available by the authors, without undue reservation.
